# Geriatric Nutritional Risk Index and Survival of Patients With Colorectal Cancer: A Meta-Analysis

**DOI:** 10.3389/fonc.2022.906711

**Published:** 2022-06-30

**Authors:** Haiming Zhao, Li Xu, Peng Tang, Rui Guo

**Affiliations:** Department of Gastroenterology, Eastern Hospital, Sichuan Academy of Medical Sciences & Sichuan Provincial People’s Hospital, Chengdu, China

**Keywords:** geriatric nutritional risk index, malnutrition, colorectal cancer, survival, meta-analysis

## Abstract

**Background:**

Geriatric nutritional risk index (GNRI) is an indicator of nutritional status derived by serum albumin level and ideal body weight, which has been proposed as a predictor of prognosis for elderly population with various clinical conditions. The objective of the meta-analysis was to comprehensively evaluate the association between baseline GNRI and survival of patients with colorectal cancer (CRC).

**Methods:**

Cohort studies were identified by search of PubMed, Embase, and Web of Science databases from inception to January 05, 2022 according to the aim of the meta-analysis. A random-effect model incorporating the potential between-study heterogeneity was adopted to pool the results.

**Results:**

Nine studies including 3658 patients with CRC contributed to the meta-analysis. Results showed that CRC patients with lower GNRI at baseline had worse overall survival (OS, hazard ratio [HR] 2.39, 95% confidence interval [CI] 1.78-3.23, p<0.001; I^2 =^ 60%) and progression-free survival (PFS, HR 1.77, 95% CI 1.38-2.26, p<0.001; I^2 =^ 33%). The results were consistent in sensitivity analyses limited to elderly patients (HR for OS 2.25, p<0.001; HR for PFS 1.65, p=0.003). Subgroup analyses showed consistent results in patents with different cancer stages, and in studies with median follow-up < and ≥ 5 years (p for subgroup effects all < 0.05).

**Conclusion:**

A lower GNRI at baseline may be independent associated with poor survival outcomes of patients with CRC. Evaluating the nutritional status using GNRI may be important for risk stratification of patients with CRC.

## Introduction

Colorectal cancer (CRC) is the third most prevalent cancer, with the annually diagnosed cases of more than 1.4 million worldwide (1–3). Aging is a risk factor for CRC, and median age of patients diagnosed with CRC is 67 years (4, 5). With the accelerated aging of the people all over the world, particularly in some developing countries, CRC will continuously be a significant threat to the health of the global population (6). On the other hand, although multiple modalities have been applied in the treatment of CRC, such as the surgical resection and radio-chemotherapies, the prognosis of patients with CRC remain poor (7, 8). Accordingly, identification of prognostic factor in patients with CRC, particularly in the elderly patients, is pivotal for the optimization of the management of these patients.

Accumulating evidence confirmed that malnutrition is a common hallmark of patients with cancer, resulting in unintentional weight loss due to a lack of intake or uptake of nutrients (9). Malnutrition has been suggested to adversely affect several aspects of cancer treatment and outcome, including reducing treatment intensity, increasing treatment toxicities, impairing patients’ quality of life, and ultimately worsening their survival (9). Indeed, pretreatment nutritional status has been related with the prognosis of patients with cancer (10). It has been suggested that the nutritional status not only affects the tolerance of the patient to the anticancer treatments (11, 12), but may also determine the response of the patients to these therapies (13). In patients with CRC, the prevalence of CRC varied between 20% and 50% according to previous reports, depending on the study population and the tools used for nutritional assessment (9, 14). For elderly patients or those advanced cancer, malnutrition, even anorexia-cachexia syndrome (ACS) occur at the diagnosis of the cancer, which could significantly compromise the overall functional status of the patients (15). Accordingly, development of reliable nutritional assessment tool is important for the clinical management of patients with malignancies, including CRC.

Previous studies have proposed several nutritional scoring systems for the evaluation of nutritional status of people with various clinical conditions, such as the malnutrition inflammation score (16), the P-POSSUM score (17), and the subjective global assessment (18). However, these evaluations are extremely complex and require the inclusion of many items. The Mini Nutritional Assessment (MNA), based on 18 questions, is recommended by the European Society of Parenteral and Enteral Nutrition to assess the nutritional status of elderly people (19). Moreover, the Nutritional Risk Score 2002 (NRS-2002), another commonly used nutritional screening tool, is calculated from three nutritional parameters including weight loss, low food intake, body mass index, and disease severity (20). However, the MNA and NRS-2002 may be misinterpreted for patients unable to provide an accurate and credible self-assessment, owing to the reliance on subjective assessment. Recently, the geriatric nutritional risk index (GNRI), a newly developed indicator of nutritional status retrieved by serum albumin concentration and ratio between actual and ideal body weight (21), has been associated with sarcopenia and frailty, two recognized risk factors of poor prognosis in older people (22, 23). The GNRI was firstly developed by Bouillanne et al. in 2005 (21) and validated as a reliable prognostic nutritional index for elderly patients with various clinical conditions, such as those admitted to a geriatric rehabilitation care unit (21), with acute ischemic stroke (24), heart failure (25), respiratory failure (26), after emergency surgeries (27). Compared with the above nutritional assessment parameters, such as MNA and NRS-2002, the GNRI is a simple, objective, and less time-consuming tool, which could also be readily determined from routinely collected laboratory data. Further studies in oncology showed that GNRI may also be applied as an effective prognostic index in patients with various malignancies, not limited to elderly patients (28). Indeed, a lower GNRI has been associated with poor prognosis in patients with various malignancies, such as those with esophageal cancer (29), renal cell carcinomas (30), and non-small cell lung cancer (31). However, the influences of GNRI on survival outcomes in patients with CRC, particularly of patients with different cancer stages, remain not comprehensively evaluated. Therefore, we performed a meta-analysis to systematically evaluate the prognostic role of GNRI in patients with CRC.

## Materials and Methods

The Preferred Reporting Items for Systematic Reviews and Meta-Analyses (PRISMA) statement (32, 33) and the Cochrane’s Handbook (34) guideline was followed in the conceiving, conducting, and reporting the study.

### Search of Databases

Studies were retrieved by search of the electronic databases including PubMed, Embase, and Web of Science from the inception of the database to January 12, 2022, with a combined search term as (“geriatric nutritional risk index” OR “GNRI”) AND (“colorectal” OR “colorectum” OR “colon” OR “rectal” OR “rectum”) AND (“neoplasms” OR “carcinoma” OR “cancer” OR “tumor” OR “malignancy” OR “adenoma”). The search was restricted to human studies published in full-length articles with no limitation of the publication language. The reference lists of the relevant original and review articles were also manually screened for possible related studies.

### Study Inclusion and Exclusion Criteria

We formulated the inclusion criteria according to the aim of the meta-analysis, with the recommended PICOS criteria.

**P (patients)**: Adult patient with CRC.
**I (exposure)**: patients with malnutrition risk as evidenced by the lower GNRI at baseline.
**C (control)**: patients without malnutrition risk as evidenced by the higher GNRI at baseline. GNRI was calculated by the following equation, as previously defined: GNRI = [1.489 × serum albumin (g/dl)] + [41.7 × actual weight/ideal weight] (21). Ideal weight was calculated using body mass index (BMI): ideal weight = 22 × (height [m]) ^2^. The cutoffs for the analyses of GNRI were consistent with the values adopted in the original studies.
**O (outcomes)**: compared the relative risk of overall survival (OS) and/or progression-free survival (PFS) between CRC patients with lower versus higher GNRI. We defined OS as the time elapsed from treatment and to the date of death from any cause and RFS as the interval between initiation of the treatment and the first recurrence or progression event.
**S (study design)**: cohort studies, including prospective and retrospective cohorts.

Reviews, preclinical studies, studies including patients with other malignancies, studies not evaluating GNRI, or studies not reporting outcomes of interest were excluded.

### Data Collection and Quality Assessing

The literature search, data collection, and study quality assessment were independently conducted by two authors separately. If discrepancies occurred, the corresponding author was contacted for discussion and reaching the consensus. We collected data regarding study information, patient demographic factors, cancer stage and treatment, GNRI cutoffs, follow-up duration, and outcomes reported for each of the included studies. Study quality was assessed *via* the Newcastle–Ottawa Scale (35) with scoring regarding the criteria for participant selection, comparability of the groups, and the validity of the outcomes. The scale ranged between 1-9 stars, with larger number of stars presenting higher study quality.

### Statistical Analyses

The relative risk for the survival outcomes, including OS and PFS, between CRC patients with lower versus higher GNRI was presented as hazard ratios (HRs) and the confidence intervals (CIs). Using the 95% CIs or p values, data of HRs and the standard errors (SEs) could be calculated, and a subsequent logarithmical transformation was conducted to keep stabilized variance and normalized distribution. Between study heterogeneity was estimated with the Cochrane’s Q test and the I^2^ statistic (36), with I^2^ > 50% reflecting the significant heterogeneity. A random-effect model was applied to combine the results by incorporating the influence of heterogeneity (34). Sensitivity analyses limiting to elderly patients (aged 65 years or above) were performed (37). Subgroup analyses were performed to evaluate the influence of cancer stage and follow-up duration on the outcomes. Medians of the continuous variables were used to define subgroups. By construction of the funnel plots, the publication bias was estimated based on the visual judgement of the symmetry of the plots, supplemented with the Egger’s regression asymmetry test (38). The RevMan (Version 5.1; Cochrane Collaboration, Oxford, UK) software package was applied for these analyses.

## Results

### Literature Search

**Figure 1:**
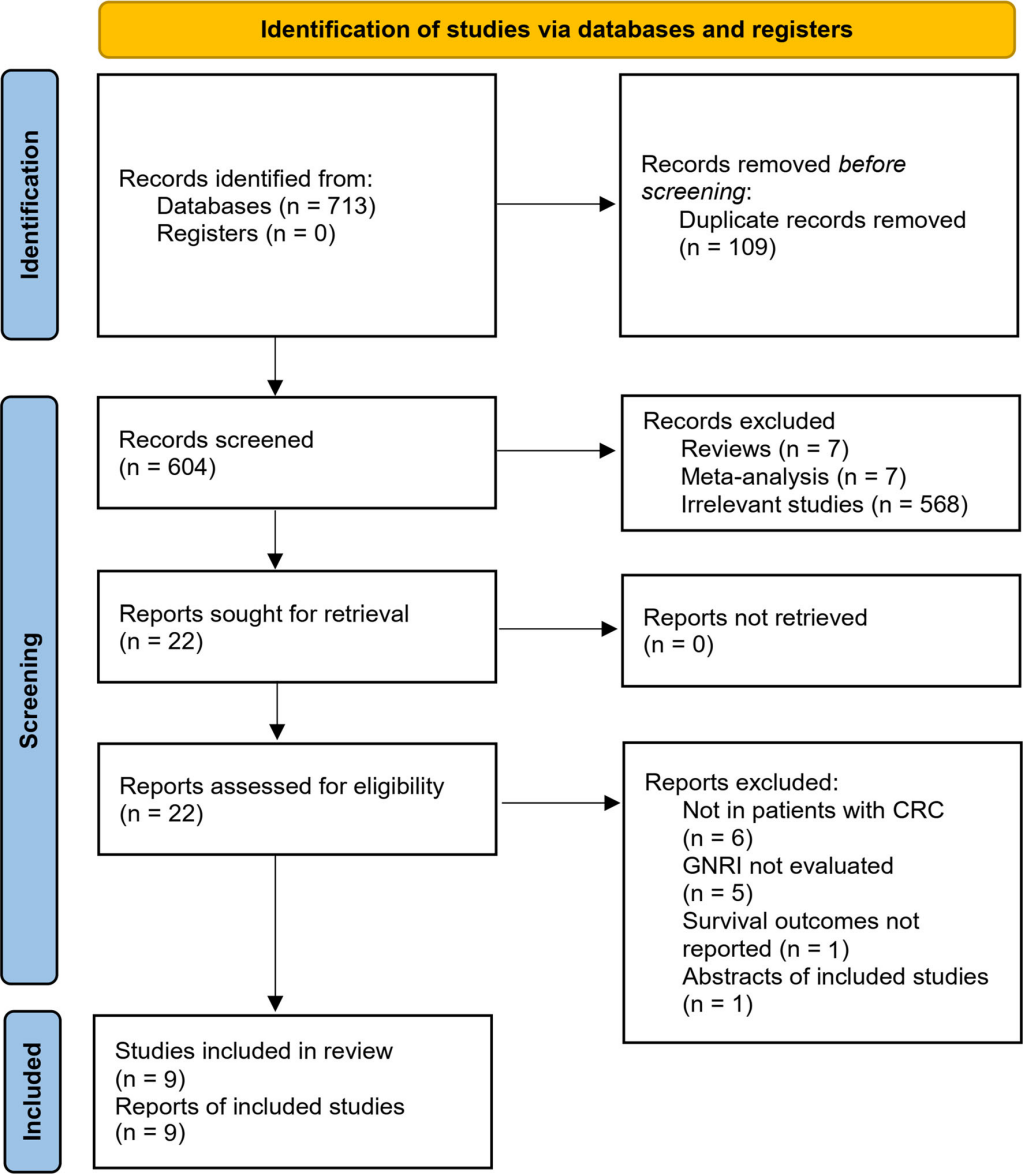
PRISMA 2020 diagram of database search and study inclusion.

The flowchart of literature search and study inclusion was displayed in **Figure 1**. In summary, 604 records were obtained in the initial database search after removing the duplications. Subsequently, 582 studies were further removed after screening with titles and abstracts, largely because they were not relevant to the objective of the meta-analysis. Finally, 22 studies underwent full-text review, and 13 were excluded for the reasons listed in **Figure 1**, which eventually made 9 studies available for the meta-analysis (39–47).

### Study Characteristics

Overall, nine retrospective cohort studies (39–47) including 3658 patients with CRC contributed to the meta-analysis. One study included two cohorts of patients with CRC (40), which were separately analyzed in the meta-analysis, making a total of ten cohorts available. **Table 1** shows the main features of the included studies. These studies were published between 2020 and 2022, and performed in Japan (39, 40, 42, 45–47) and China (41, 43, 44). The cancer stage of the included patients varied from stage I to stage IV, and the treatments mainly included surgical resection, endoscopic submucosal dissection, and chemoradiotherapy. The cutoffs for defining of the lower versus higher GNRI were also varied among the included studies, and were mostly derived from the receiver operating characteristics (ROC) curve analysis (40–44, 46, 47). The median follow-up durations varied from 2.6 to 5.5 years. All of the ten cohorts reported the outcome of OS, while the outcomes of PFS were reported in six cohorts (39, 41–43, 45, 46). Multivariate analyses were applied to present the association between GNRI and survival of RCR in all of the included studies, and confounding factors including age, sex, performance status, cancer location, stage, and treatment etc. were adjusted among the original studies. The NOS of the included studies were 7 to 8 stars, suggesting generally good study quality (**Table 2**).

**Table 1:** Characteristics of the included cohort studies.

Study	Country	Design	Sample size	Diagnosis	Mean age (years)	Men (%)	Cancer stage	Treatment	GNRI cutoff	Median follow-up (years)	Outcomes reported	Variables adjusted
Iguchi 2020 (39)	Japan	RC	80	CRC with liver metastasis	63.6	55	IV	Surgery	≤98 vs >98	4.2	OS and PFS	Age, sex, CRC location, lymphomatic metastasis, perioperative chemotherapy, and procedure features
Tang 2020 (41)	China	RC	230	CRC patients aged≥65 years	70.6	70	I-IV	Surgery	≤98 vs >98 (ROC derived)	5.1	OS and PFS	Age, sex, tumor stage, perineural/vascular invasion, pathological type, surgical approach, and CEA
Sasaki 2020 (40)	Japan	RC	313	CRC patients aged≥65 years	73	64.2	I-IV	Surgery	≤98 vs >98 (ROC derived)	5.1	OS	Age, sex, BMI, WBC, CRP, biomarkers, tumor stage
Sasaki 2020 (40)	Japan	RC	218	CRC patients aged≥65 years	72	60.6	I-IV	Surgery	≤98 vs >98 (ROC derived)	5.5	OS	Age, sex, BMI, tumor biomarkers, location, and stage
Liao 2021 (43)	China	RC	1206	CRC patients aged≥75 years	80.5	55.8	I-III	Surgery	≤98 vs >98 (ROC derived)	5.1	OS and PFS	Age, sex, BMI, CCI, WBC, tumor location, tumor stage, pathological type, and procedural features
Ruan 2021 (44)	China	RC	201	Patients with CRC	72	65.5	I-IV	Surgery and radio- or chemotherapy	≤92 vs >92 (ROC derived)	3.7	OS	Age, sex, radical resection, tumor stage, PS, comorbidities and treatments
Ide 2021 (42)	Japan	RC	93	Patients with local advanced rectal cancer	63	73	I-III	Surgery and radio- or chemotherapy	≤104 vs >104 (ROC derived)	5	OS and PFS	Age, sex, pathological type, tumor stage, and characteristics of treatments
Doi (45) 2022	Japan	RC	329	Patients with CRC	73.5	45	I-III	Surgery	≤98 vs >98	2.6	OS and PFS	Age, sex, and tumor stage
Hayama 2022 (46)	Japan	RC	259	CRC patients aged≥65 years	74.2	55.6	I-III	Surgery	≤101 vs >101 for OS, ≤91 vs >91 for PFS (ROC derived)	3.3	OS and PFS	Age, sex, BMI, tumor location, pathological type, tumor stage, and tumor biomarkers
Kato 2022 (47)	Japan	RC	729	CRC patients aged≥75 years	79.2	58.2	I	Endoscopic submucosal dissection with or without surgery	≤96 vs >96(ROC derived)	3.6	OS	Age, sex, PS, CCI, history of malignancy, and different procedures

GNRI, geriatric nutritional risk index; RC, retrospective cohort; CRC, colorectal cancer; ROC, receiver operating characteristics; OS, overall survival; RFS, recurrence-free survival; CEA, carcinoembryonic antigen; BMI, body mass index; WBC, while blood cell; CRP, C-reactive protein; CCI, Charlson Comorbidity Index; PS, functional status.

**Table 2:** Details of study quality evaluation *via* the Newcastle-Ottawa Scale.

Study	Representativeness of the exposed cohort	Selection of the non-exposed cohort	Ascertainment of exposure	Outcome not present at baseline	Control for age	Control for other confounding factors	Assessment of outcome	Enough long follow-up duration	Adequacy of follow-up of cohorts	Total
Iguchi 2020 (39)	0	1	1	1	1	1	1	1	1	8
Tang 2020 (41)	0	1	1	1	1	1	1	1	1	8
Sasaki 2020 (40)	0	1	1	1	1	1	1	1	1	8
Sasaki 2020 (40)	0	1	1	1	1	1	1	1	1	8
Liao 2021 (43)	0	1	1	1	1	1	1	1	1	8
Ruan 2021 (44)	0	1	1	1	1	1	1	1	1	8
Ide 2021 (42)	0	1	1	1	1	1	1	1	1	8
Doi 2022 (45)	0	1	1	1	1	1	1	0	1	7
Hayama 2022 (46)	0	1	1	1	1	1	1	1	1	8
Kato 2022 (47)	0	1	1	1	1	1	1	1	1	8

### GNRI and OS of Patients With CRC

**Figure 2:**
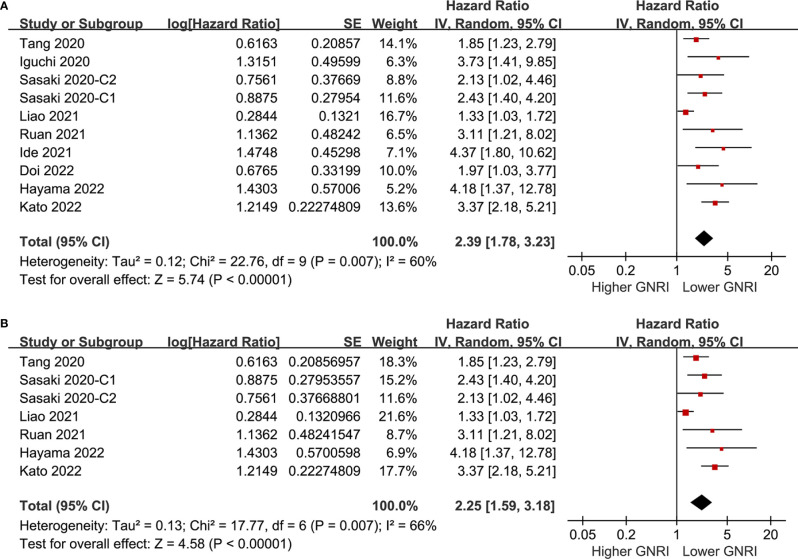
Forest plots for the meta-analysis regarding the association between GNRI and OS in patients with CRC; **(A)** overall meta-analysis; and **(B)** sensitivity analysis limited to the elderly patients.

**Figure 3:**
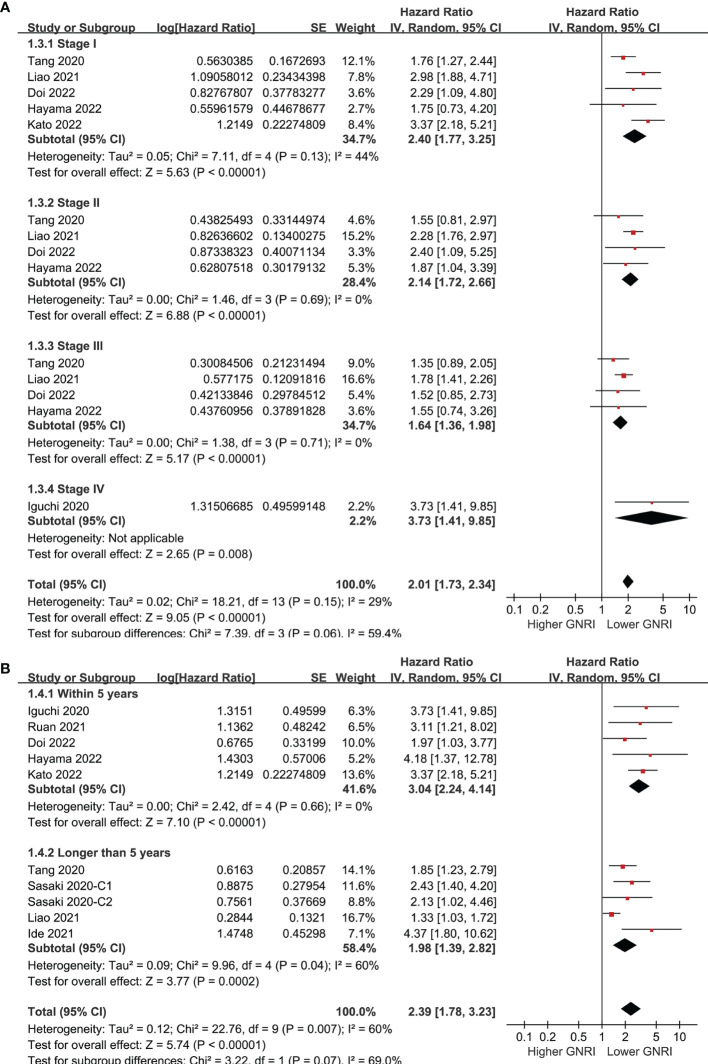
Forest plots for the subgroup analysis regarding the association between GNRI and OS in patients with CRC; **(A)** subgroup analysis according to the stage of cancer; and **(B)** subgroup analysis according to the follow-up duration.

Pooling the results of ten cohorts (39–47) showed that compared to those with higher GNRI, a lower GNRI at baseline was associated with worse OS in patients with CRC (HR 2.39, 95% CI 1.78-3.23, p<0.001; I^2 =^ 60%; **Figure 2A**). Sensitivity analysis limited to elderly patients showed consistent results (HR 2.25, 95% CI 1.59-3.18, p<0.001; I^2 =^ 66%; **Figure 2B**). Subgroup analyses showed that a lower GNRI was associated with poor OS in patients with stage I (HR 2.40, 95% CI 1.77-3.25, p<0.001), stage II (HR 2.14, 95% CI 1.72-2.66, p<0.001), stage III (HR 1.64, 95% CI 1.36-1.98, p<0.001), and stage IV (HR 3.73, 95% CI 1.41-9.85, p=0.008; **Figure 3A**) CRC, and in studies with follow-up duration < 5 years (HR 3.04, 95% CI 2.24-4.14, p<0.001) and ≥ 5 years (HR 1.98, 95% CI 1.39-2.82, p<0.001; **Figure 3B**).

### GNRI and PFS of Patients With CRC

**Figure 4:**
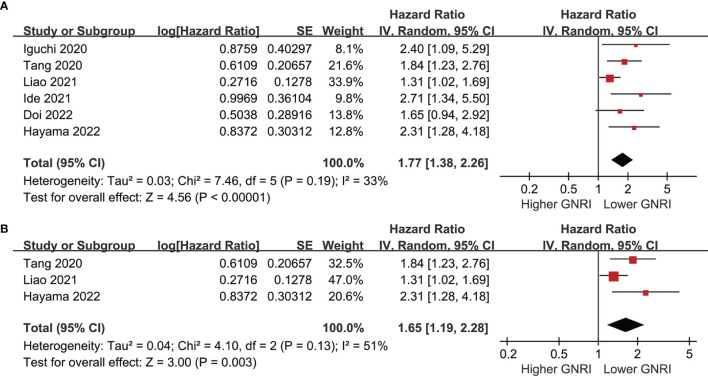
Forest plots for the meta-analysis regarding the association between GNRI and PFS in patients with CRC; **(A)** overall meta-analysis; and **(B)** sensitivity analysis limited to the elderly patients.

**Figure 5:**
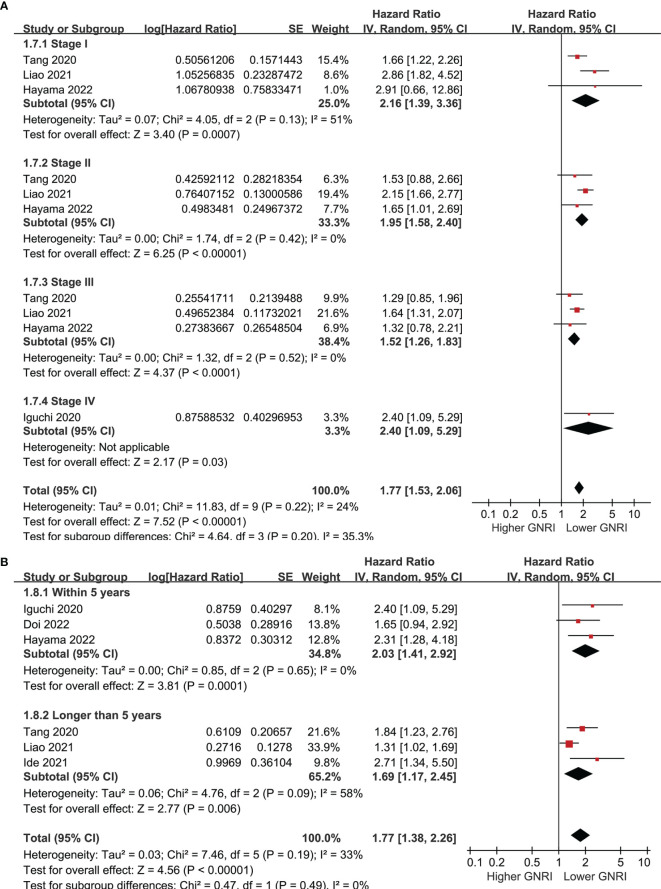
Forest plots for the subgroup analysis regarding the association between GNRI and PFS in patients with CRC; **(A)** subgroup analysis according to the stage of cancer; and **(B)** subgroup analysis according to the follow-up duration.

Six cohorts (39, 41–43, 45, 46) evaluated the association between GNRI and PFS of CRC. Results showed that a lower GNRI at baseline was also associated with worse PFS in patients with CRC (HR 1.77, 95% CI 1.38-2.26, p<0.001; I^2 =^ 33%; **Figure 4A**), and the association remains in elderly patients (HR 1.65, 95% CI: 1.19-2.28, p=0.003; I^2 =^ 51%; **Figure 4B**). Further subgroup analyses showed similar results in patients with stage I-IV CRC (HR 2.16, 1.95, 1.52, and 2.40, p all<0.05; **Figure 5A**), and in studies with follow-up duration < 5 years (HR 2.03, p<0.001) and ≥ 5 years (HR 1.69, p=0.006; **Figure 5B**).

### Publication Bias

**Figure 6:**
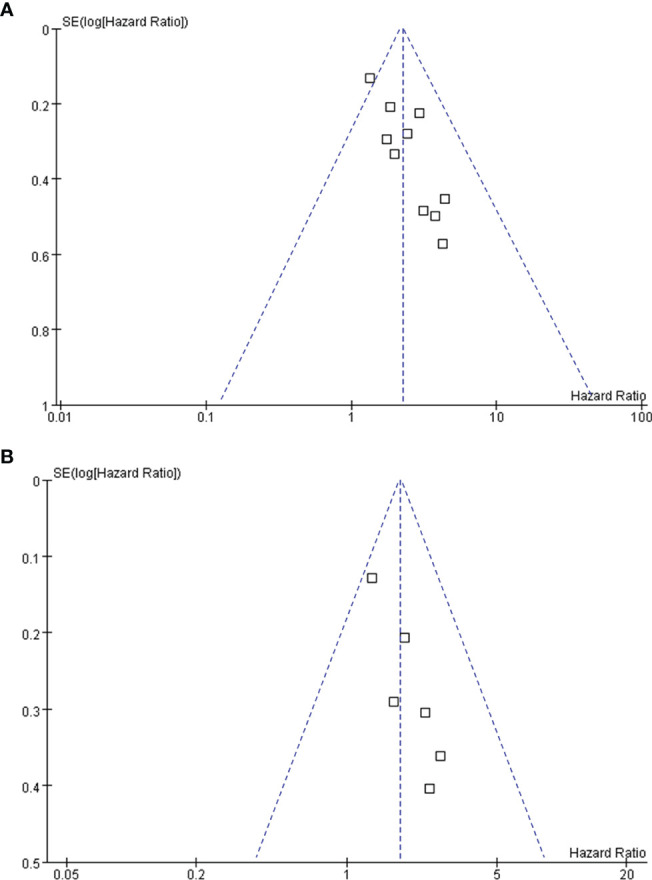
Funnel plots for the publication bias underlying the meta-analyses; **(A)** funnel plots for the meta-analysis of OS; and **(B)** funnel plots for the meta-analysis of PFS.

**Figures 6A, B** display the funnel plots for the outcomes of OS and PFS. Visual inspection revealed symmetry of the plots, reflecting a low risk of publication biases. The Egger’s regression tests also indicated low risk of publication biases (P = 0.22 and 0.14, respectively).

## Discussion

By pooling the results of ten cohorts from nine studies, this meta-analysis showed that a lower GNRI at baseline was independently associated with poor survival outcomes in patients with CRC, as evidenced by the results of both OS and PFS. Further results of sensitivity analyses showed that the association between lower GNRI and poor survival in patients with CRC was consistent in the elderly patients. Moreover, subgroup analyses showed consistent results in patients with different cancer stages, and in studies with follow-up durations < and ≥ 5 years. Taken together, these findings suggested that a lower GNRI at baseline may be an independent predictor of poor survival outcomes of patients with CRC. Evaluating the nutritional status with GNRI may be important for risk stratification of the CRC patients.

To the best of our knowledge, this may be the first meta-analysis regarding the predictive role of GNRI for survival in patients with CRC. Although some previous meta-analyses have suggested that GNRI may be applied as a prognostic factor for patients with cancer (28, 48), patients with malignancies from different sites of the body were included. The possible differences of disease course and treatments for the various malignancies may lead to significant heterogeneity, making the result difficult to be interpreted (28, 48). In this study, we included patients with CRC only, and showed that a lower GNRI at baseline was significantly associated poor survival outcomes in these patients. The strengths of this meta-analysis included the following. First, an up-to-date literature search was performed, which retrieved nine cohort studies published between 2020 and 2022 to reflect the most recent evidence regarding the predictive role of GNRI for survival of CRC. Second, all the included studies were cohort studies, which therefore could indicate a longitudinal association between lower baseline GNRI and poor survival of CRC. Third, multivariate analyses were applied among all of the included cohort studies when the association between GNRI and CRC were presented. Accordingly, results of the meta-analysis suggest the association between lower GNRI and poor survival of CRC was independent of possible confounding factors, such as age, sex, tumor location, and cancer stage etc. Finally, subsequent sensitivity analyses limited to elderly patients and subgroup analyses according to the cancer stage and follow-up durations showed consistent results, which further confirmed the robustness of the finding.

The GNRI is a convenient tool which could be easily calculated based on the serum albumin, height, and body weight of the patients. Results of our meta-analysis support the use of GNRI as a feasible and efficient prognostic tool for patients with CRC. From our point of view, the mechanisms underlying the association between GNRI and survival in patients with CRC could be explained by the roles of the components of GNRI in patients with cancer. A lower serum albumin (49) has been associated with poor survival of patients with cancer. Indeed, albumin has been involved in multiple anticancer processes of the body and related treatments, such as the maintaining osmotic pressure (50), delivering bioactive anticancer molecules (51), inhibition of overactivated inflammation (52), modulation of immune response (53), and anti-oxidative stress (54). On the other hand, compared to patients with normal weight, both overweight and underweight patients with CRC were associated with poor OS and PFS, as indicated by a previous meta-analysis of 18 observational studies (55). Moreover, a recent cohort study including 902 patients with stage II-III CRC who were treated with surgical resection showed a U-shape curve for the relationship body weight and mortality risk, suggesting that both weight loss and excessive weight gain being detrimental in these patients (56). Finally, a lower preoperative GNRI has been associated with an increased risk of severe postoperative complications in patients with various gastrointestinal malignancies (57), which may also partly explain the association between GNRI and survival of CRC.

Results of the meta-analysis suggest that identification of CRC patients with malnutrition by GNRI allows the providers to stratify patients at risk of poor survival. In addition, the results also highlight the importance of nutritional support as a direct consequence of the malnutrition assessment in CRC patients. Once it is established that patients have a low GNRI, a prompt nutritional support should be provided. In fact, regardless of the specific results of this meta-analysis, it has been generally recommended that early nutritional support in cancer patients, especially in those with gastrointestinal malignancies, could serve a complementary intervention to active treatments (58, 59). An adequate nutritional support could positively affect the tolerance to therapies, care plan continuity, quality of life and survival outcomes (60).

There are also some limitations of the meta-analysis. Firstly, all the studies were performed in East Asia, and results of the meta-analysis should be validated in studies from other countries. In addition, the optimal cutoff value for the predictive efficacy of GNRI in patients with CRC remains to be determined, and a dose-response relationship between GNRI and CRC remains to be established. Large prospective cohort studies are needed in this regard. Moreover, it is important to determine if the side (right or left), histological type, and the cancer molecular features of CRC would affect the predictive role of GNRI for the survival outcomes in patients with CRC. However, we were unable to determine the influences of these variables because subgroup data on the above tumor characteristics were not reported in the included studies. Future studies are warranted for further investigation. Besides, GNRI was only measured for once among the included studies. The clinical significance of repeated evaluation of GNRI and its influence on the choice of anticancer should be determined in future studies, too. Finally, as a meta-analysis of observational studies, we could not exclude other factors that may affect the association between GNRI and survival outcomes in patients with CRC, such as dietary factors or nutritional interventions that may affect the serum albumin levels.

In conclusion, results of the meta-analysis suggest that a lower GNRI at baseline may be independent associated with poor survival outcomes of patients with CRC. Evaluating the nutritional status using GNRI may be a convenient and efficient way to improve the risk stratification of patients with CRC.

## Data Availability Statement

The original contributions presented in the study are included in the article/supplementary material. Further inquiries can be directed to the corresponding author.

## Author Contributions

HZ convinced the study. HZ and LX performed literature search, data collection, and study quality evaluation. HZ, PT, and RG performed statistical analyses and interpreted the results. HZ drafted the manuscript. All authors revised the manuscript and approved the submission.

## Conflict of Interest

The authors declare that the research was conducted in the absence of any commercial or financial relationships that could be construed as a potential conflict of interest.

## Publisher’s Note

All claims expressed in this article are solely those of the authors and do not necessarily represent those of their affiliated organizations, or those of the publisher, the editors and the reviewers. Any product that may be evaluated in this article, or claim that may be made by its manufacturer, is not guaranteed or endorsed by the publisher.
